# Species-specific prophage induction by ciprofloxacin in human gut metagenomes

**DOI:** 10.1128/msystems.00303-26

**Published:** 2026-08-24

**Authors:** Ben Sakdinan, Anshul Sinha, Firdausi Qadri, Ashraful I. Khan, Eric J. Nelson, B. Jesse Shapiro

**Affiliations:** 1 Department of Microbiology & Immunology, McGill University 549337, Montréal, Quebec, Canada; 2 McGill Centre for Microbiome Research, Montréal, Quebec, Canada; 3 Infectious Diseases Division, International Centre for Diarrhoeal Disease Research, Bangladesh 56291, Dhaka, Bangladesh; 4 Emerging Pathogens Institute, University of Florida 145775, Gainesville, Florida, USA; Universidad de Los Andes, Bogota, Colombia

**Keywords:** prophage induction, antibiotics, metagenomics, gut microbiome, microbial ecology, bacteriophages

## Abstract

Antibiotics are known to trigger prophage induction in controlled laboratory settings, but it remains unclear whether this also occurs within microbiomes in nature. Current methods investigating the link between antibiotics and prophage induction within the human gut rely on *in vitro* culturing of human gut bacterial isolates. Using a metagenomic approach, we aimed to measure prophage induction, and whether it is associated with antibiotic exposure. In two independent human cohorts, we compared prophage to bacterial host read depth ratios (P:H) across known or measured antibiotic exposures. We found that induction is not broadly associated with antibiotic exposures at the level of the overall microbiome, but that ciprofloxacin increases P:H ratios in certain bacterial species. We documented heterogeneous trajectories of P:H ratios over the course of antibiotic exposure, sometimes increasing and remaining high, or returning to baseline. This study complements experimental models by providing *in vivo* evidence of induction in the human gut.

**IMPORTANCE:**

Bacteriophages are viruses that infect a bacterial host. The lytic and lysogenic cycles are the two classic outcomes of phage infection. In the lytic cycle, the phage immediately replicates and lyses its host cell to release new viral particles. In the lysogenic cycle, the phage, now called a prophage, integrates its genome into that of its host without killing it. Prophages can switch to the lytic cycle in a process called induction, in which the viral genome is replicated, the host cell is lysed, and viral particles are released. The most immediate consequence of induction is host cell death, which can impact bacterial populations and communities. Since prophages are mobile genetic elements that can move between bacteria, they are also an important vehicle for horizontal gene transfer. While induction has been well studied *in vitro*, whether and how induction occurs within the complex microbial ecosystem in humans is less well characterized. Understanding prophage induction *in vivo* is therefore critical in corroborating *in vitro* observations.

## OBSERVATION

Current studies investigating the triggers of prophage induction employ *in vitro* or animal models (1), with little research directly studying the causes of induction within microbiomes. For example, ciprofloxacin triggers DNA damage and the SOS response (2), resulting in prophage induction *in vitro* (3–6). Yet, evidence of prophage induction occurring in natural settings, such as the human gut microbiome, is lacking. Here, we aim to address this knowledge gap by inferring prophage induction in stool metagenomic data from two human cohorts with varying levels of antibiotic exposure: diarrheal patients from Bangladesh with antibiotic exposures quantified in their stool by mass spectrometry, and healthy American (U.S.) volunteers sampled before and after a course of ciprofloxacin (Supplemental text). Using these data sets, we calculated the prophage to bacterial host read depth ratio (henceforth abbreviated to P:H ratio) as a measure of induction.

## NO EVIDENCE FOR SYSTEMATIC COMMUNITY-WIDE PROPHAGE INDUCTION BY ANTIBIOTICS

**Fig 1:**
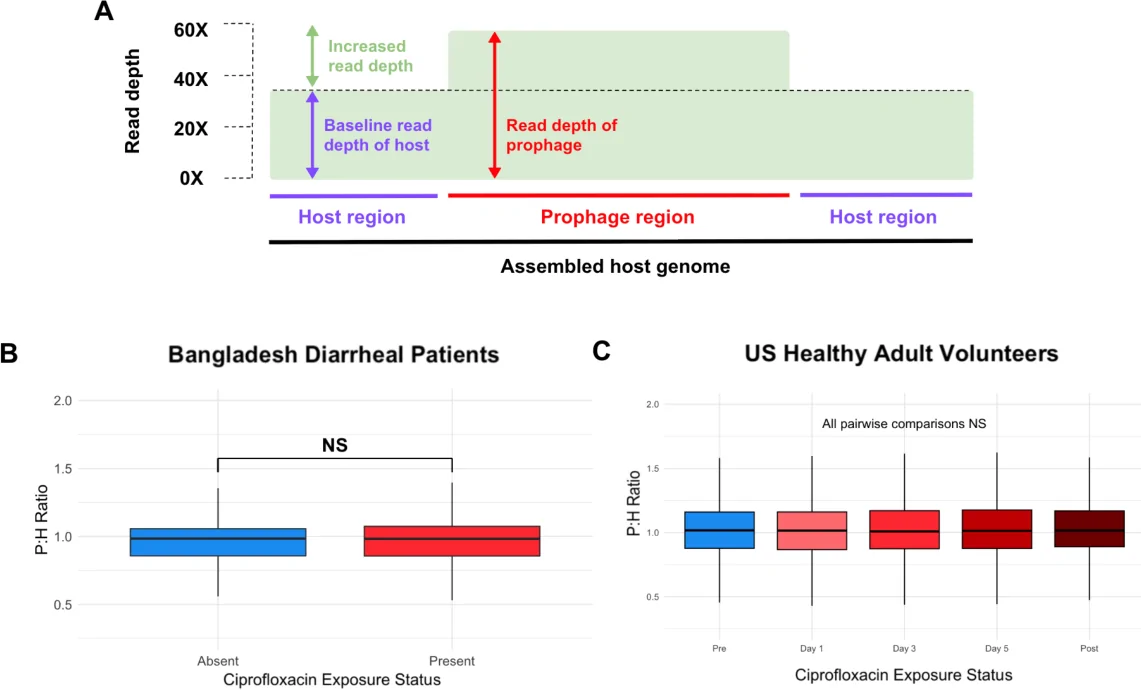
Quantification of prophage induction in human gut microbiomes across antibiotic exposures using metagenomic read mapping. (**A**) Conceptual visualization of the prophage to host (P:H) ratio as calculated from metagenomic reads mapped to assembled contigs containing prophages. (**B**) P:H ratio across ciprofloxacin exposure status in the Bangladesh cohort. Statistical significance was assessed with a Wilcoxon rank-sum test. (**C**) P:H ratios in healthy U.S. volunteers before (pre), during (days 1, 3, and 5), and after (post) ciprofloxacin exposure. Statistical significance was assessed with a Wilcoxon rank-sum test; NS indicates not significant (*P* > 0.05).

To test the hypothesis that antibiotics systematically induce prophages in the gut microbiome, we mapped metagenomic reads to virus-containing contigs to measure the ratio of read depth of the prophage region to the host region. When a prophage is induced, its genome is replicated and released from the host cell as newly assembled virions. Such relative changes in genome copy number are captured by the P:H ratio (Fig. 1A; Supplemental methods) (7).

We estimated the P:H ratio in 4,055 prophages from the Bangladesh cohort and assessed their association with measured antibiotic exposures (Table S1). We set the following thresholds for exposure: ciprofloxacin (0.063 µg/mL), doxycycline (0.13 µg/mL), and azithromycin (8.0 µg/mL). Based on our previous work, these exposures are detectable but not necessarily inhibitory for bacterial growth (8). We found no significant increase in community-wide P:H ratios in the presence of ciprofloxacin (Fig. 1B) or other antibiotics. Azithromycin exposure was associated with a slight decrease in P:H ratios from a median of 1.0 to 0.98, potentially due to selection of bacterial genomes without an integrated prophage (Fig. S1) (9). As an additional test of our hypothesis in a healthy cohort, we calculated P:H ratios in 15,677 prophages assembled from United States healthy adult volunteers (Table S1) and compared the ratios at timepoints before, during, and after a controlled ciprofloxacin exposure. We found no significant difference in the P:H ratios from baseline (pre-exposure) to any post-antibiotic time point (Fig. 1C). Together, the analysis of both cohorts indicates that antibiotic exposure is not measurably associated with broad-scale prophage induction in gut metagenomes.

## CIPROFLOXACIN IS ASSOCIATED WITH PROPHAGE INDUCTION IN SPECIFIC BACTERIAL SPECIES

**Fig 2:**
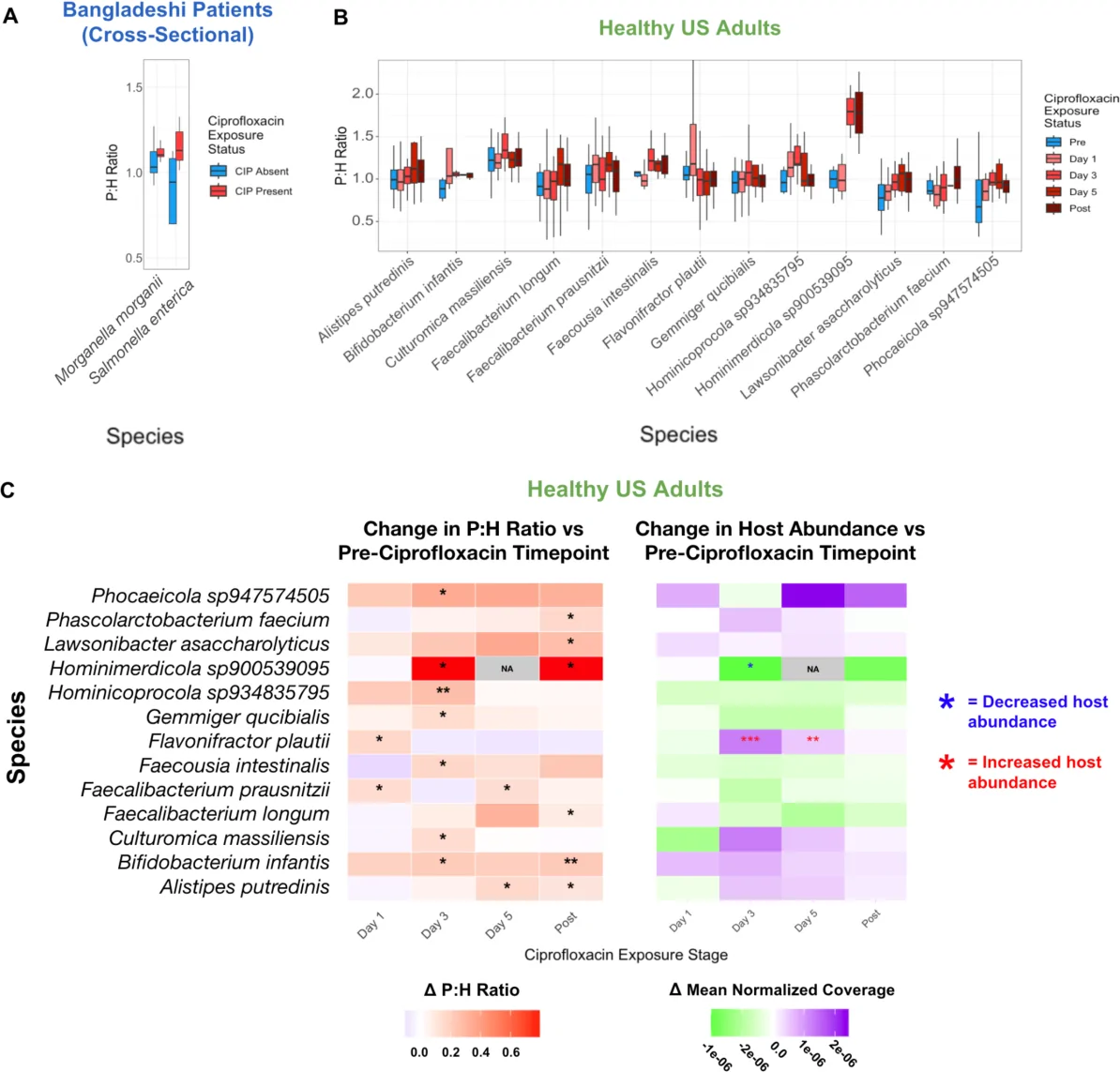
Ciprofloxacin exposure is significantly correlated with increased P:H ratios in multiple prophage host species. (**A**) Species in the cross-sectional Bangladesh cohort with a significant increase in P:H ratio between patients exposed vs. not exposed to ciprofloxacin. Statistical significance was assessed with a Wilcoxon rank-sum test with Bonferroni multiple correction. Only species with corrected *P* ≤ 0.05 are shown. (**B**) Species in the U.S. healthy cohort with a significant increase in P:H ratios between baseline and a post-ciprofloxacin exposure time point. Only species with a Bonferroni corrected Wilcoxon rank-sum test *P*-value ≤ 0.05 at one or more time points are shown. (**C**) Heatmap showing the same species as in panel B. In the left panel, cells are colored based on the difference (delta) between the P:H ratio at the given timepoint compared with baseline (pre-ciprofloxacin exposure). In the right panel, changes in host relative abundance were assessed by measuring the depth of coverage of the prophage host, and normalizing by total reads in the sample. Bonferroni-adjusted Wilcoxon rank-sum test *P-*values are coded as follows: NS, *P* > 0.05; *, *P* ≤ 0.05; **, *P* ≤ 0.01.

While we found no evidence for measurable antibiotic-induced prophage induction at the overall community level, we hypothesized that induction could be restricted to specific taxa. To investigate this, we classified prophages by their predicted host taxonomy, and clustered them at the species or genus levels. In the Bangladesh cohort, we considered 19 prophages with an inferred bacterial host species that were present in at least 20 patients (Fig. S2). Consistent with an experimental study (5), we found that ciprofloxacin exposure was associated with an increase in the median P:H ratio in *Salmonella enterica* from 0.94 to 1.13, and an increase in *Morganella morganii* from 1.02 to 1.09 (Wilcoxon rank-sum, *P* < 0.05 after a conservative Bonferroni multiple test correction). In both species, P:H ratios increased from ~1 in the absence of ciprofloxacin exposure to >1 with detectable ciprofloxacin (Fig. 2A). We detected no instances of significant decreases in P:H ratios with antibiotic exposure. More than one genetically distinct prophage was identified per host species in most patients, suggesting that these effects are driven by more than a single prophage per species. Induction is more likely driven by ciprofloxacin than by differences in patient diarrheal disease status (Fig. S3).

Diarrheal patients in Bangladesh commonly self-medicate with antibiotics; hence, the exact timing of antibiotic exposure in these patients is unknown. Antibiotic concentrations were only measured at the time of stool sample collection. To address this uncertainty, we next considered the cohort of healthy U.S. adults in which the timing of ciprofloxacin was known precisely (10). In these healthy U.S. volunteers, we considered 176 prophages matched with a host species and present in at least 20 patients. By comparing each post-exposure timepoint to their pre-exposure baseline, we found that ciprofloxacin exposure was significantly correlated with increased P:H ratios in at least one time point in 13 bacterial species (Fig. 2B) and decreased P:H ratios in five species (Fig. S4). Decreasing P:H ratios could be due to antibiotics selecting against lysogens relative to bacteria not containing prophages, reducing rates of induction, or both. Selection against lysogens may be explained by increased energy costs of harboring a prophage, yielding additive or compounding fitness costs under antibiotic stress (11). Increasing P:H ratios could also be explained by free phage particles persisting while abundance of the host significantly decreases. To address this possibility, we tested for decreases in host relative abundance at each timepoint, as compared with the pre-exposure baseline. Host abundance was approximated using sequencing depth normalized by total read count per sample. Across all species, we observed only a single significant decrease, in *Hominimerdicola* sp900539095 (Fig. 2C). The absence of consistent decreases across other taxa suggests that elevated P:H ratios are not generally attributable to reductions in host abundance, and are more likely driven by induction. The most common peak of induction was observed on the third day of ciprofloxacin exposure, with seven species having a significant difference at this timepoint (Wilcoxon rank-sum test, *P* < 0.05 after Bonferroni correction). Induction persisted at the post-exposure timepoint in six species (Fig. 2B and C).

We further hypothesized that increased P:H ratios would lead to increased host bacterial lysis, reducing host relative abundance at subsequent timepoints. However, species with significantly increased P:H ratio at one post-exposure timepoint had no tendency to decline in relative abundance at subsequent timepoints compared with the background distribution of species without evidence for induction (Kolmogorov-Smirnov test, *P* > 0.05). This finding is consistent with induction having only mild effects on host bacterial population sizes (12, 13).

## PERSPECTIVES

We report evidence of species-specific prophage induction by ciprofloxacin in the human gut. The P:H ratios we measured are in line with other recent theory and metagenomic estimates (12, 13) and lower than *in vitro* burst sizes generating ratios of 100 or more (14). A variety of factors could explain this relatively low-level induction in the gut. Bacteria could experience lower antibiotic concentrations in the gut than typically applied in the laboratory. P:H ratios could also be suppressed when not all host genomes encode a prophage. An important caveat is that P:H ratios could be increased if antibiotics kill bacteria while free viral particles persist at stable abundances. Our interpretation of elevated P:H ratios as evidence for induction may therefore not be universal, but is a likely explanation given that ciprofloxacin is a known inducer of *Salmonella* and other bacteria under laboratory conditions. We only observed significant signals of induction with exposure to ciprofloxacin and not other antibiotics. This is expected, as ciprofloxacin triggers the bacterial SOS response through DNA damage (15), the canonical trigger of prophage induction (2). Doxycycline and azithromycin do not have known DNA damaging activity. Regardless of the explanation, it is clear that antibiotics are not strong or universal inducers of prophages in the gut, but they likely have subtle, species-specific effects on P:H ratios. The generality of these results across antibiotic classes and host bacteria remain to be seen, along with potential consequences on microbiome structure and function.

## SUPPLEMENTAL MATERIAL

Supplemental figures and tables msystems.00303-26-s0001.docx Table S1 and Figures S1-S4.

Supplemental text msystems.00303-26-s0002.pdf Methodology and brief discussion of supplemental results.

STORMS checklist msystems.00303-26-s0003.docx STORMS checklist for gut microbiome research.

ASM does not own the copyrights to Supplemental Material that may be linked to, or accessed through, an article. The authors have granted ASM a non-exclusive, world-wide license to publish the Supplemental Material files. Please contact the corresponding author directly for reuse.
