# Supplementary material for: Species-specific prophage induction by ciprofloxacin in human gut metagenomes
Source: mSystems. 2026 Aug 24;11(9):e00303-26. doi: 10.1128/msystems.00303-26 (PMC13595984; doi:10.1128/msystems.00303-26)
Supplement: Supplementary file 1 [file msystems.00303-26-s0002.pdf]

Supplementary Material

Methods

Datasets. Publicly available sequencing data from our previous study was used to assemble 344 metagenomes from stool samples that were collected from cholera patients enrolled in hospitals across Bangladesh between March to April 2019.[1] These metagenomic libraries all received DNA from a bacteria-enriched pellet and phage-enriched supernatant prior to sequencing. Dehydration severity for each patient was assessed and recorded as mild, moderate, or severe. This dataset contains concentrations of ciprofloxacin, doxycycline, and azithromycin, as measured by liquid chromatography mass spectrometry. Short reads for 344 cholera patients were accessed from our previous study by Madi et al. 2024,[1] available in the NCBI Short Read Archive under BioProject PRJNA976726.

In our second dataset we analyzed publicly available metagenomes from Yaffe et al.[2] This dataset contains metagenomic reads sequenced from stool samples collected from 60 healthy adults over 20 weeks surrounding a 5-day exposure event to ciprofloxacin. Participants took 500mg of ciprofloxacin twice daily during these 5 days. For each of the 60 patients in this longitudinal study, data from 5 timepoints were selected: 3 and 0 days before ciprofloxacin exposure (baseline), days 3, and 5 during ciprofloxacin exposure, and 5 days post exposure. Reads were downloaded from NCBI under BioProject PRJNA974858.

The STORMS checklist for microbiome research is provided in the supplemental materials.

Metagenomic Data Analyses. Short reads for Bangladeshi diarrheal patient metagenomes and healthy US adult volunteers were assembled using MEGAHIT v1.2.9.[3] Assembled contigs less than 1kb were removed with SeqKit v2.5.1.[4] Prophage contigs were predicted with geNomad v1.8.1.[5] Quality and completeness of prophage contigs were assessed using CheckV1.0.3.[6] Prophage to host read ratios were calculated using PropagAtE v1.1.0.[7] Prophages were clustered into viral operational taxonomic units (vOTUs) based on shared gene content using VContTACT2 v0.11.3.[8] Probable prophage hosts were predicted using iPHoP v1.3.3.[9]

Host Relative Abundance Calculations. To calculate the relative abundance of a prophage's host species and genus, we measured the depth of coverage of contigs assigned to those hosts and normalized these values by the total read count in the sample (total sequencing depth). This normalization accounts for scenarios where a host genome is covered more deeply due to sequencing effort. This method thus measures the abundance of the host relative to the total library size.

To determine if host species with significant signals of prophage induction tend to decline in relative abundance at subsequent time points, we calculated the change in each prophage host's relative abundance between each timepoint. This analysis was only completed within the US cohort, since time-series sampling was not available in our Bangladesh cohort. We subset the host species into those 13 in which we detected significantly increased P:H ratios in, and all other hosts as a non-induced background. We used the Kolmogorov-Smirnov test to determine if the changes in relative abundances within our subset of significantly induced hosts significantly deviate from that of non-induced hosts.

Statistical Analyses. All statistical analyses and generation of plots was conducted using R, version 2023.12.1+402 (2023.12.1+402).

To test for significant differences in P:H ratios between antibiotic exposure statuses, distributions of P:H ratios were compared using the Wilcoxon rank-sum test (Mann Whitney U test). In the case where multiple comparisons were made, such as in testing multiple host species, Bonferroni multiple test correction was performed. *P*-values were assigned with the following scheme: $P > 0.05$ = NS, $P \leq 0.05$ = *, $P \leq 0.01$ = **, $P \leq 0.001$ = ***.

To test if a host species declined in relative abundance, normalized depths of coverage for each host species at each timepoint were compared to their baseline (day 0) values using Bonferroni adjusted Wilcoxon rank-sum test. *P*-values are coded as: NS: $P > 0.05$, *: $P \leq 0.05$, **: $P \leq 0.01$.

To test if host species tended to decline in relative abundance at the subsequent time points following a significant increase in P:H ratio, the difference in relative abundance between each timepoint was calculated for each of these species. Next, we subset these host species into those that had a significant signal of prophage induction, and those that did not. The distributions

of relative abundance differences were compared between these two subsets using the Kolmogorov-Smirnov test.

Code Availability. Example code used in this project is available on GitHub: https://github.com/bensakdinan/Discovering-phages-from-metagenomes.git

Supplementary Results

Prophage induction is primarily driven by ciprofloxacin, not disease-related stress

We recognize that the Bangladesh cohort suffered from diarrheal disease, potentially causing stress to the microbiome that could also modulate prophage induction. We previously found that antibiotic exposure is associated with milder dehydration severity in this cohort.[1] The median P:H ratio of *Salmonella enterica* prophages was increased by 21% in patients with mild dehydration (**Figure S3**). It is unlikely that mild dehydration is causing induction, but rather that antibiotics are driving both induction and milder disease. Consistent with this hypothesis, among the 13 prophages found in patients with mild dehydration, all were exposed to ciprofloxacin, while only two of the 11 prophages in patients with non-mild dehydration were exposed to ciprofloxacin (Fisher's exact test, OR > 5, $P = 4.2e\text{-}5$). These results suggest that ciprofloxacin exposure, not dehydration status, is the dominant factor contributing to prophage induction in diarrheal patients.

Supplemental References

1. Madi, N. *et al.* Phage predation, disease severity, and pathogen genetic diversity in cholera patients. *Science* **384**, eadj3166.

2. Yaffe, E. *et al.* Brief antibiotic use drives human gut bacteria towards low-cost resistance. *Nature* **641**, 182–191 (2025).

3. Li, D., Liu, C.-M., Luo, R., Sadakane, K. & Lam, T.-W. MEGAHIT: an ultra-fast single-node solution for large and complex metagenomics assembly via succinct de Bruijn graph. *Bioinforma. Oxf. Engl.* **31**, 1674–1676 (2015).

4. Shen, W., Sipos, B. & Zhao, L. SeqKit2: A Swiss army knife for sequence and alignment processing. *iMeta* **3**, e191 (2024).

5. Camargo, A. P. *et al.* Identification of mobile genetic elements with geNomad. *Nat. Biotechnol.* https://doi.org/10.1038/s41587-023-01953-y (2023) doi:10.1038/s41587-023-01953-y.

6. Nayfach, S. *et al.* CheckV assesses the quality and completeness of metagenome-assembled viral genomes. *Nat. Biotechnol.* **39**, 578–585 (2021).

7. Kieft Kristopher & Anantharaman Karthik. Deciphering Active Prophages from Metagenomes. *mSystems* **7**, e00084-22 (2022).

8. Bin Jang, H. *et al.* Taxonomic assignment of uncultivated prokaryotic virus genomes is enabled by gene-sharing networks. *Nat. Biotechnol.* **37**, 632–639 (2019).

9. Roux, S. *et al.* iPHoP: An integrated machine learning framework to maximize host prediction for metagenome-derived viruses of archaea and bacteria. *PLoS Biol.* **21**, e3002083 (2023).
