# Supplementary material for: Species-specific prophage induction by ciprofloxacin in human gut metagenomes
Source: mSystems. 2026 Aug 24;11(9):e00303-26. doi: 10.1128/msystems.00303-26 (PMC13595984; doi:10.1128/msystems.00303-26)
Supplement: Supplementary file 2 [file msystems.00303-26-s0001.docx]

**Supplementary Table**

**Supplementary Table 1.** **Overview of metagenomic assembly and viral contig identification**. Each row shows the number of contigs in each of the two datasets (columns) at each stage of assembly and annotation.

**Supplementary Figures**

## **Supplementary figure 1. Signals of prophage induction across exposure to antibiotics.** (A) P:H ratio vs doxycycline exposure status within Bangladesh diarrheal patients. (B) P:H ratio vs azithromycin exposure status within Bangladesh diarrheal patients. (C) P:H ratio vs any antibiotic exposure status within Bangladesh diarrheal patients. Statistical significance was assessed with a Wilcoxon rank-sum test; NS: *P* > 0.05, *: *P* ≤ 0.05.

## **Supplementary figure 2. All species tested for induction in the Bangladesh dataset.** P:H ratios vs ciprofloxacin exposure status in prophages clustered by their predicted host, within Bangladeshi diarrheal patients. To be included in this plot, a given species must have been assembled from at least 20 patients and contain a datapoint at both statuses of ciprofloxacin exposure. Statistical significance calculated by Wilcoxon rank-sum with Bonferroni multiple correction NS: *P* > 0.05, *: *P* ≤ 0.05.

**Supplementary figure 3.** **In Bangladeshi diarrheal patients, mild dehydration is significantly correlated with increased P:H ratio for *Salmonella enterica* prophages**. P:H ratios vs dehydration severity in prophages clustered by their predicted host, within Bangladeshi diarrheal patients. Statistical significance calculated by Wilcoxon rank-sum with Bonferroni multiple correction NS: p > 0.05, *:*P* ≤ 0.05, **: *P* ≤ 0.01.

**Supplementary figure 4.** **Ciprofloxacin exposure is significantly correlated with decreased P:H ratio in multiple prophage host species**. Species in the US healthy cohort with a significant decrease in P:H ratios between baseline and a post-ciprofloxacin exposure time point. Only species with a Bonferroni corrected Wilcoxon rank-sum test *P*-value ≤ 0.05 at one or more time points are shown.
